# Suppression of superconductivity at the nanoscale in chemical solution derived YBa_2_Cu_3_O_7−*δ*_ thin films with defective Y_2_Ba_4_Cu_8_O_16_ intergrowths†

**DOI:** 10.1039/d0na00456a

**Published:** 2020-06-24

**Authors:** Ziliang Li, Mariona Coll, Bernat Mundet, Anna Palau, Teresa Puig, Xavier Obradors

**Affiliations:** Institut de Ciència de Materials de Barcelona, CSIC, Campus de la UAB 08193 Bellaterra Catalonia Spain xavier.obradors@icmab.es

## Abstract

The analysis of the microstructure and superconducting behavior of chemical solution deposited epitaxial YBa_2_Cu_3_O_7−*δ*_ films, with thickness going down to 5 nm has been carried out with the purpose to disclose the behavior of the most common intergrowth in these films, the Y_2_Ba_4_Cu_8_O_16_. The analysis of ultrathin films is a unique opportunity to investigate the superconducting behavior of these nanoscale defects because of the high concentration created as a consequence of the elastic energy associated to the misfit strain. Magnetic susceptibility and X-ray diffraction measurements evidence a strong decrease of the superconducting volume correlated with an increase of the intergrowth volume fraction. We demonstrate that these intergrowths are non-superconducting nanoscale regions where Cooper pair formation is disrupted, in agreement with their key role as artificial pinning centers for vortices in YBa_2_Cu_3_O_7−*δ*_ films and coated conductors.

## Abstract 2

The analysis of the microstructure and superconducting behavior of chemical solution deposited epitaxial YBa_2_Cu_3_O_7−*δ*_ films, with thickness down to 5 nm, has been carried out to disclose the behavior of the most common intergrowth Y_2_Ba_4_Cu_8_O_16_.

## Graphical abstract

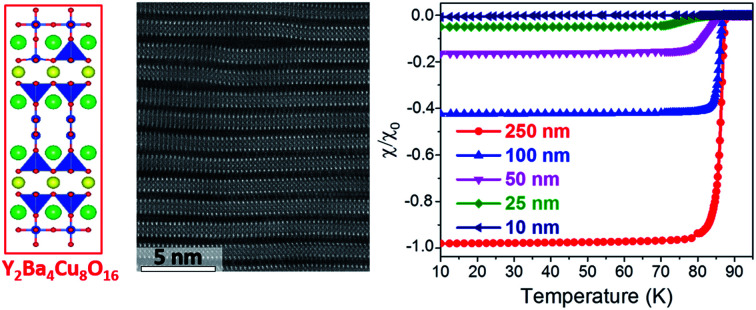

## 1. Introduction

Since the discovery of high temperature superconductors (HTS) there has been an intensive analysis of the complex relationship between lattice structure, defects and superconducting properties.^1^ This correlation, however, becomes even more relevant when one intends to develop high current superconducting tapes and wires for power applications where the defect structure strongly influences the most relevant superconducting parameter, *i.e.* critical current density *J*_c_(*H*,*T*).^2^ After having achieved epitaxial films and coated conductors (CCs), *i.e.* HTS films grown on biaxially buffered metallic substrates not influenced by grain boundary disorder, the main issue has become to understand which defects behave as effective artificial pinning centers (APC) of vortices and hence increase *J*_c_(*H*,*T*) at high temperatures and magnetic fields.^3,4^ Vortex pinning in HTS is actually a complex research topic because one needs to correlate the intricate physical behavior of vortices with the defect landscape of these materials in order to disclose how the different defects contribute to pin vortices at different temperatures, magnetic fields and field orientations.^5–9^

In the case of YBa_2_Cu_3_O_7−*δ*_ (Y123) films and CCs extensive analyses have been carried out to sort out how to enhance vortex pinning. Particularly, huge progress has been achieved with the development of several processing approaches to produce nanocomposite films where secondary nanometric non-superconducting phases coexist with the Y123 matrix.^7–13^ A very relevant issue in this field has been to disclose how the Y123 matrix is modified by the secondary phases, such as the perovskites BaZrO_3_, BaHfO_3_, Ba_2_YTaO_6_, or other non-superconducting oxides, and how they influence vortex pinning.^7,8,10,12,14,15^ These secondary phases leads to the formation of Y_2_Ba_4_Cu_8_O_16_ (Y248) intergrowths having an extra Cu–O layer^16^ so extensive efforts to disclose its influence on vortex pinning has been undertaken.^3,9,10^

Chemical solution deposition (CSD) has been demonstrated to be a very attractive technique for large-scale production of Y123 films and CCs owing to its cost effectiveness advantage.^17,18^ CSD is a prototypical example of Y123 epitaxial growth where secondary phases in the form of nanoparticles can be easily included.^12,15,19–23^ They usually remain randomly oriented, generating a high interfacial energy which partially relaxes through the formation of induced defects such as the Y248 intergrowths.^10,24^ Particularly, in CSD nanocomposites it has been shown that vortex pinning is strongly enhanced by the concentration of these Y248 intergrowths.^9,10,25^

The structure of the planar Y248 intergrowth defects has been recently analyzed in detail by Scanning Transmission Electron Microscopy (STEM). The expected composition of the Y248 intergrowth is Y_2_Ba_4_Cu_8_O_16_ because they consist on an extra Cu–O chain layer inserted between two Ba–O layers, leading to a lattice expansion in *c*-axis direction (from 11.7 to 13.3 Å).^16,26^ Considering the stoichiometric ratio for a pristine Y123 film (*i.e.* Y : Ba : Cu = 1 : 2 : 3), the formation of these intergrowths has been recently described as double chains including defect clusters formed by two Cu vacancies decorated by three O vacancies.^27^ The stoichiometric Y248 phase (without vacancies) is superconducting with a lower *T*_c_^28^ than the Y123 phase. However, the superconducting performance of the Cu off-stoichiometric Y248 phase is still unknown because these planar defects are dispersed within CSD Y123 films, as it was shown in previous reports,^10,19^ and hence it becomes very difficult to sort out their superconducting properties. Actually, recent studies have shown, on one hand, that ferromagnetic clusters are formed around the Cu vacancies defects in the double chains and so one should wonder if this causes a pair breaking effect.^27,29^ On the other hand, it has also been noticed that the defective double chains induce distortions at the nanoscale, in the neighboring CuO_2_ planes, and oxygen vacancies and also generate highly strained localized areas at the partial dislocations surrounding the Y248 intergrowth,^23^ as detected by atomic scale STEM studies.^30,31^ Both of them may induce Cooper pair breaking effects.^30^ In order to have a better understanding of the superconducting performance of this type of defective Y248 intergrowths it is crucial to grow Y123 epitaxial films with a high concentration of them.

In this paper, we present an analysis of the micro/nanostructure and superconducting properties of Y123 epitaxial films of different thicknesses grown by CSD where a high concentration of Y248 intergrowths is developed when ultrathin films are formed. Y123 films with thickness down to 5 nm have been successfully fabricated using an optimized CSD growth process allowing to reach such small thickness keeping a high film homogeneity. Actually, ultrathin Y123 films have been recently used as seed layer to improve the epitaxial quality of nanocomposite films and this provided some hints that a high concentration of Y248 could be achieved.^10,20,21,23^ Our purpose here is to maximize this opportunity to depict the influence of this microstructural defect on the superconducting properties. We show first by X-ray diffraction and Scanning Transmission Electron Microscopy (STEM) that, indeed, a high concentration of Y248 intergrowths is achieved in Y123 ultrathin films. We show then that these defects have a remarkable influence on the superconducting properties of the films. Particularly, we discern a linear relationship between the Y248 intergrowth concentration and the superconducting volume, determined from magnetic shielding measurements. Our results allow to infer the non-superconducting character of the Y248 intergrowths observed in Y123 thin films.^32–35^

## 2. Experimental section

The Y123 precursor solution was prepared by the reaction from solid Y123 ceramic powders (yttrium–barium–copper oxide, Solvay) with trifluoroacetic anhydride as described in detail elsewhere.^36^ The original obtained anhydrous precursor solutions were diluted from 1.5 M to 0.3–0.03 M using anhydrous methanol for the purpose of achieving an adjustment of film thicknesses, ranging from 250 nm to 5 nm. After depositing the solutions on 5 × 5 mm^2^ commercially available (100) LAO or STO single-crystal substrates by spin-coating at a typical rotation speed of 6000 rpm for 2 min, the low-temperature (310 °C) pyrolysis process^37^ in a humid oxygen atmosphere was conducted to prepare solid precursor films. We have found that an improved film quality is achieved on LAO substrates where lattice mismatch induces an in-plane compressive strain to Y123 (*f*^LAO^ = (*a*^LAO^ − *a*^Y123^)/*a*^Y123^ = −1.6%), as compared to STO substrates which induce an in-plane tensile strain to Y123 (*f*^STO^ = +1.3%) (see ESI†). Therefore, our main analysis was performed on films grown on LAO substrates.

In the following crystallization step, an optimized thermal process was necessary to be developed to achieve ultrathin films with enough good quality (see ESI†). Flash Heating (FH) is a novel recently developed process based on high heating ramps (∼30 times faster than in conventional thermal annealing (CTA), *i.e.* ∼750 °C min^−1^), thus leading to a reduced total heating time ≤1 min.^23,25^

A series analysis of several processing parameters, *e.g.* heating process, crystallization temperature and thermal annealing time, were carried out to avoid dewetting effects in ultrathin films. It is known that dewetting effects are promoted at long annealing times and high temperature annealing.^38^ To minimize the detrimental dewetting effects we used FH. The optimal annealing temperature was found at 810 °C. At lower temperatures the films have a tendency to include large pores and misoriented grains. At higher temperatures, some secondary phases are identified by SEM-EDX and X-ray diffraction. The crystallization stage was performed during 20 min in a wet N_2_-0.02% O_2_ mixed gas atmosphere with a water partial pressure (P(H_2_O)) of 23 mbar which was introduced at 110 °C. After that, an extra 10 min dwell was proceeded at the crystallization temperature in dry N_2_-0.02% O_2_ gas to minimize film imperfections which generated from the grain boundary zipping, misfit strain, porosity, *etc.*^18,39^ Reasonably smooth ultrathin films with uniform film thickness (5–15% variation depending on thickness) were only achieved using FH and short enough annealing times, otherwise film dewetting was originated, as evidenced by SEM, TEM and AFM images (see ESI†). Finally, the oxygenation process of the well crystallized and grown films were performed at 550 °C for 3 h in dry oxygen atmosphere.

Film surface morphology was studied by scanning electron microscopy (SEM) in a planar view and Atomic force microscopy (AFM) analysis on tapping mode with a molecular imaging system. AFM images were processed and analyzed with the commercial software package MOUNTAINS (Digital Surf); see Fig. S7 and S8.†

The phase analysis and texture characterization of the fully converted Y123 ultrathin films were carried out by two-dimensional X-ray diffraction (XRD) patterns using a Bruker AXS GADDS diffractometer. As a supplement for the limited resolution of the GADDS system, a high-resolution XRD (HRXRD) *θ*–2*θ* scan using a Bruker-AXS (model A25 D8 Discover) X-ray diffractometer was also applied for the phase identification. Non-uniform r.m.s. strain (nanostrain) (*ε*) was determined using the Williamson–Hall (WH) method^40,41^ by analyzing the symmetric (00*l*) 2*θ* Bragg diffraction integral breadth *β* acquired in a Siemens D5000 diffractometer. The fitting was made following the following equation:
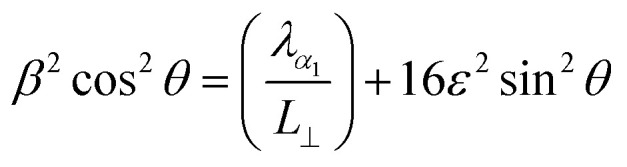
where *θ* is the Bragg angle, *λ*_*α*_1__ is the wavelength of the Cu K_α_ radiation and *L*_⊥_ is the size of the coherent volume perpendicular to the scattering vector (*c*-axis in our case). Nanostrain *ε* corresponds, therefore, to the disorder in (00*l*) plane separation along the *c*-axis.

In-plane and out-of-plane texture analysis were analyzed from the (103) Y123 phi-scan (*ϕ*-scan) and (005) Y123 rocking curve (*ω*-scan), respectively. The microstructural characteristics of Y123 ultrathin films were described by scanning transmission electron microscopy (STEM) using a FEI Titan 60–300 microscope equipped with an X-FEG gun, a CETCOR probe corrector and a Gatan TRIDIEM 866 ERS energy filter operated in STEM mode at 300 kV. Superconducting properties were investigated from magnetization measurements performed with a superconducting quantum interference device (SQUID) magnetometer (Quantum Design, San Diego, CA) equipped with a 7 T magnet. Low field (∼0.2 mT) Zero Field Cooled (ZFC) temperature dependent magnetic susceptibility measurements with *H*‖*c* were used to determine the superconducting volume, *T*_c_ and Δ*T*_c_. Critical current densities *J*_c_(*H*,*T*) with *H*‖*c* were determined from isothermal hysteretic magnetization measurements *M*(*H*,*T*) (see ESI†) or from temperature dependent remnant magnetization *M*(*T*) measurements, performed after applying and suppressing a magnetic field of 7 T to assure full field penetration, to calculate *J*^sf^_c_(*T*). The Bean model approximation to thin discs, *J*_c_(*H*,*T*) = 3*M*(*H*,*T*)/*R*, where *R* is the effective radius of the sample and *M*(*H*,*T*) is the hysteretic magnetization, was used to calculate the critical current densities.^42,43^

**Fig. 1:**
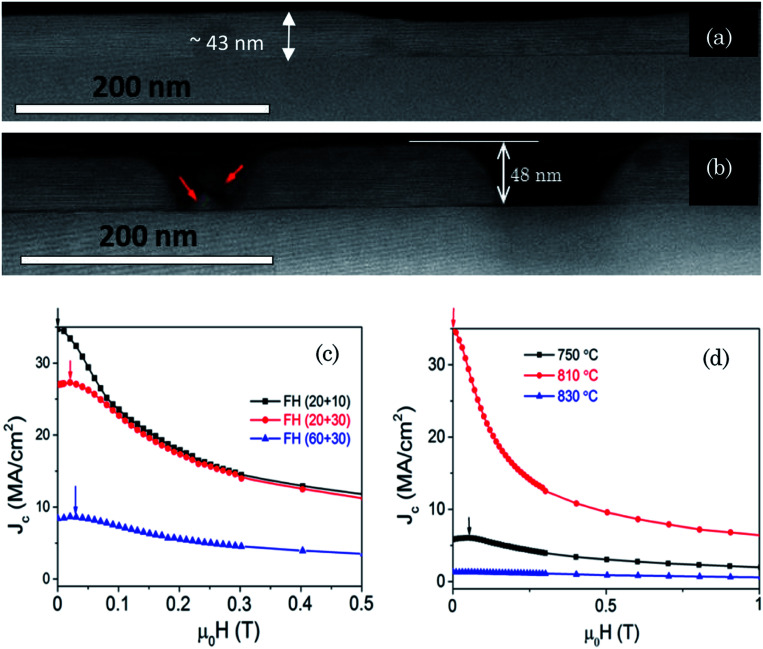
Cross sectional STEM image of 50 nm Y123 ultrathin films grown during different annealing times or temperatures (a) FH 810 °C with 20 min wet anneal and 10 min dry anneal process; (b) FH 810 °C with 60 min wet anneal and 30 min dry anneal process; (c) magnetic field dependence of the critical current density measured at 5 K for the 50 nm Y123‖LAO films grown by FH at 810 °C following different wet + dry annealing times, as indicated in the caption, (20 + 10) (black square), (20 + 30) (red circle) and (60 + 30) (blue triangle); Films having the highest critical currents correspond to those not exhibiting dewetting, while the other ones show a progressive influence of dewetting; (d) magnetic field dependence of the critical current density measured at 5 K of the 50 nm Y123/LAO films grown by FH at temperatures of 750 °C, 810 °C and 830 °C.

Optimization of the annealing process of ultrathin films was also based on the study of the isothermal magnetic field dependence of the critical current densities *J*_c_(*H*) determined from isothermal magnetization measurements (Fig. 1(c) and (d)). The isothermal critical current densities of the different thin films were estimated from the recorded isothermal magnetization hysteresis loops, using the Bean model approximation to thin films, *J*_c_(*H*,*T*) = 3*M*(*H*,*T*)/*R*, where *R* is the effective radius of the sample. The hysteretic magnetization is *M* = (*m*_p_ − *m*_n_)/*V*, calculated from the positive and negative values of magnetic moment and *V* is the volume of the film.

The large *J*_c_(*H*) values in Fig. 1(c) and (d) indicate that an improved homogeneity of the films has been achieved, *i.e.* pores and dewetting have been minimized. Also the observation of a peak in *J*_c_(*H*) at finite magnetic fields (Fig. 1(c) and (d)) has been previously attributed to granularity effects in porous thin films, *i.e.* in films exhibiting some dewetting for instance.^44–46^ Optimally grown films, instead, display a maximum in *J*_c_(*H*) at zero external magnetic field.

## 3. Results and discussion

### 3.1. Structural characterization of the films

#### a X-ray diffraction study

**Fig. 2:**
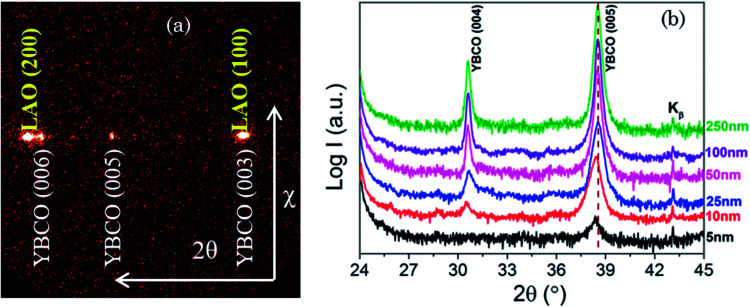
(a) A typical two-dimensional XRD frame of Y123 film with thickness of 50 nm where only the (00*l*) Bragg reflections corresponding to epitaxial growth are identified; (b) high resolution XRD *θ*–2*θ* scans of Y123 films with different thicknesses ranging from 5 nm to 250 nm. The dotted line indicates the 2*θ* position of thick Y123 films. Note that intensity has a logarithmic scale and the patterns are vertically shifted.

**Fig. 3:**
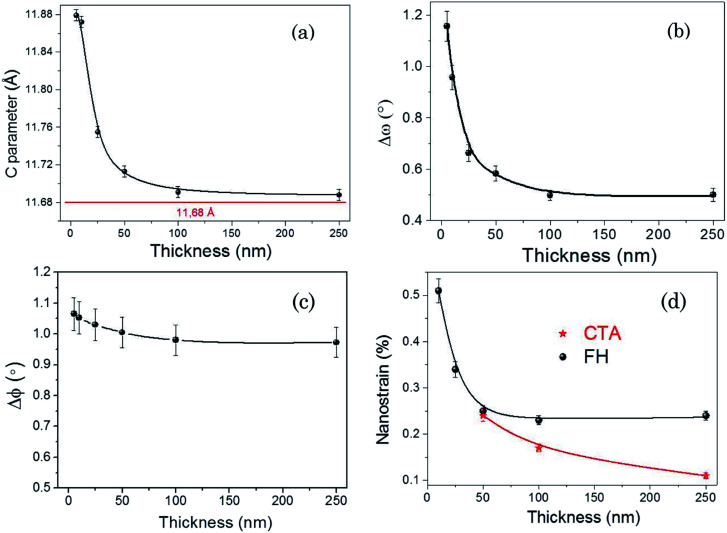
The evolution with Y123 film thickness of: (a) the *c*-axis lattice parameter. The red line corresponds to bulk Y123; (b) FWHM of *ω*-scans (Δ*ω*), (c) FWHM of *ϕ*-scans (Δ*ϕ*); (d) nanostrain *ε* measured in films prepared by Conventional Thermal Annealing (CTA) and Flash Heating (FH).

Fig. 2 displays the X-ray diffraction (XRD) analysis of pristine Y123 films with different thickness. A typical two dimensional *θ*–2*θ* XRD (GADDS) frame of pristine Y123 thin films with thickness of 50 nm is shown in Fig. 2(a). Note that solely (00*l*) diffraction poles of Y123 are identified, indicating that the films are epitaxial without any polycrystalline or randomly oriented Y123 grains. In Fig. 2(b) we present the high resolution XRD scans for films with different thickness, ranging from 5 to 250 nm. It is observed that Y123 thin films only show (00*l*) Bragg reflections, demonstrating that *c*-axis oriented Y123 grains are obtained throughout the investigated film thickness. This result suggests that *c*-oriented Y123 thin film can be obtained in an extended thickness window down to 5 nm for CSD-based Y123 thin films, in a similar thickness limitation compared with the vacuum-based deposition routes.^47–49^ All the films are pristine Y123 phase without any trace of residual secondary phases. Another detail we can note from the high resolution XRD plots (Fig. 2(b)) is the shift of the (005) Y123 peaks to smaller angles when the film thickness decreases below 25 nm, which indicates an increase in the *c*-axis lattice parameter, from 11.69 Å to 11.87 Å (Fig. 3(a)).^47^ The observed maximum *c*-axis increase appears to be consistent with the Poisson's ratio *ν* = 0.314 of Y123 in the case of having a fully coherent epitaxy with the LAO substrate.^47,50^ It's also worth to mention that the observed highest *c*-axis parameter (*c* = 11.87 Å) is larger than that of a fully oxygen deficient Y123 phase (YBa_2_Cu_3_O_6_) and so we cannot attribute this increase to an oxygen deficiency of the ultrathin film Y123 layers.^51^

In Fig. 3(b) and (c), we present the Δ*ω* and Δ*ϕ* evolution with film thickness, which gives us the estimation of texture quality in out-of-plane and in-plane direction, respectively. The Δ*ω* increases from ∼0.5° to ∼1.2° when film thickness decreases from 250 nm to 5 nm (Fig. 3(b)), revealing a decreased out-of-plane texture quality in our ultrathin films. This tendency is similar to what other authors have been previously reported for Y123 films deposited by sputtering method.^52^ Moreover, the Δ*ϕ* values were found to be constant (Δ*ϕ* = 1.0 ± 0.1°) down to the minimum CSD-based Y123 film thickness measured so far, *i.e.* 5 nm, indicating good in-plane texture quality. We also determined the evolution of nanostrain *ε* in these films and we realized that it increases in parallel with the *c*-axis expansion up to *ε* = 0.5% (see Fig. 3(d)) while at film thicknesses above 50 nm a clear enhancement of nanostrain is detected for FH films as compared to CTA films. These results point us to the conclusion that the lattice cell is strongly distorted in CSD Y123 ultrathin films on LAO. The increase of the *c*-axis parameter is due to the compressive mismatch with the substrate and, at the same time, we disclose that some disorder is generated in the periodicity along *c*-axis. Lattice increases have been extensively observed in vacuum grown Y123 ultrathin films, attributing it either to the lattice misfit induced lattice distortion^47,53,54^ or to the oxygen content changes.^55,56^ More detailed analysis of the structural disorder in FH CSD Y123 films will be described hereinafter.

#### b Transmission electron microscopy analysis

**Fig. 4:**
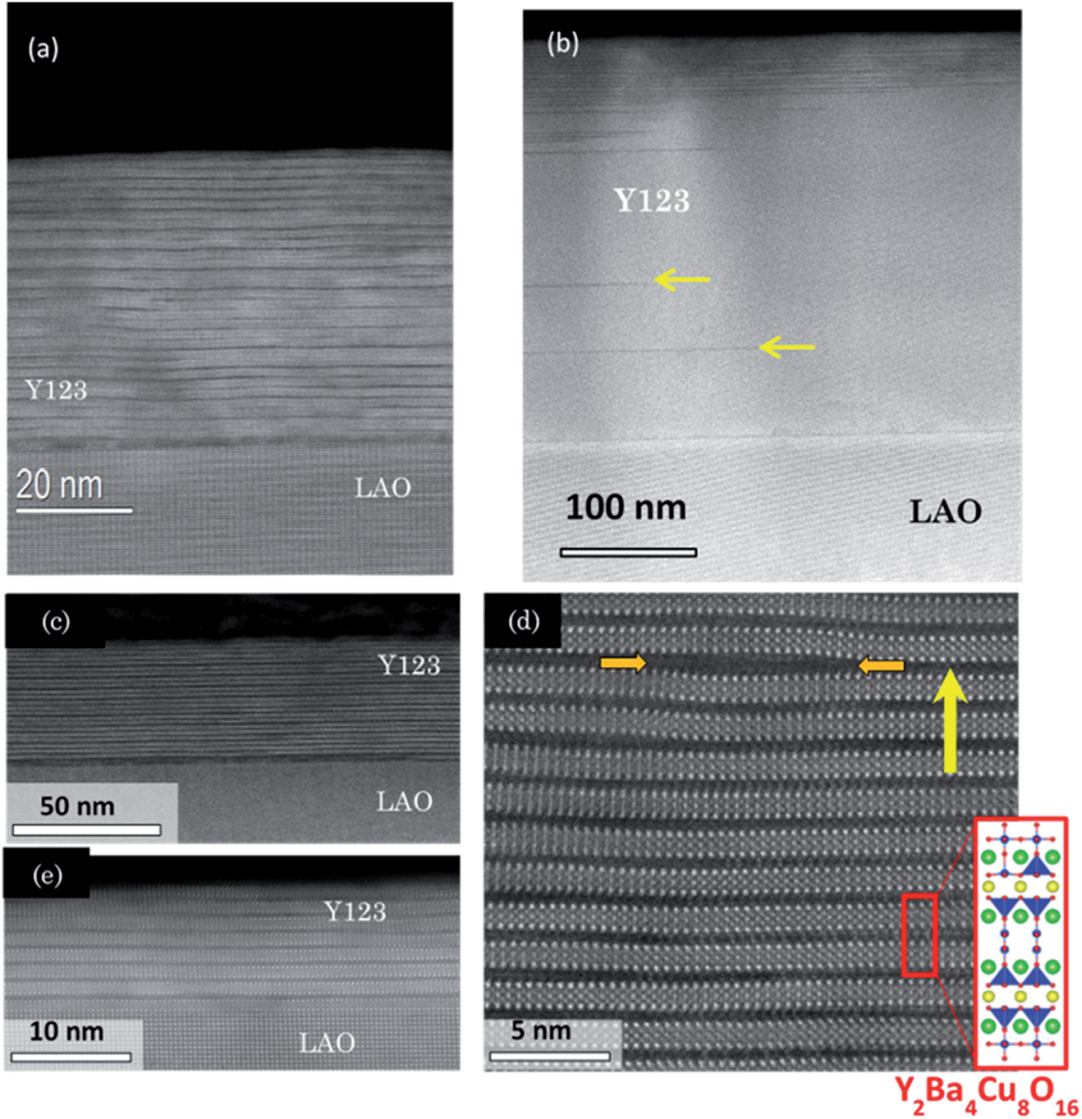
(a) STEM image of a FH Y123 film with a thickness of 45 nm where Y248 layers (black horizontal lines) are seen to be distributed inhomogeneously along *c*-axis; (b) pristine 250 nm Y123 thin film grown by the CTA process at 810 °C. The yellow arrows indicate examples of Y248 intergrowths; (c) *Z*-contrast low magnification STEM image of a 50 nm film with a high concentration of Y248 intergrowths; (d) a higher resolution STEM image of the 50 nm ultrathin film shown in (c) focusing on the bulk of the film. The inset shows the Y248 structure and its association with the observed planes; (e) *Z*-contrast STEM image of a 10 nm film. The horizontal dark stripes that cross the image are due to the formation of Y248 intergrowths. The yellow arrows in (e) show an intergrowth with an additional CuO layer resulting in the local formation of the Y125 structure.

Annular dark field (ADF) STEM imaging investigations have been conducted in order to further disclose the particular nanostructural landscape of the Y123 ultrathin films. Cross-sectional STEM images of Y123 thin films are shown in Fig. 4. The STEM images of the 45–50 and 10 nm FH Y123 thin films (Fig. 4(a), (c) and (e)) show that the Y123 film has a high density of long intergrowths (horizontal dark stripes in the image), as compared to a CTA film of 250 nm thickness (Fig. 4(b)). Note that the Y248 intergrowths can have different homogeneity distribution throughout the whole cross-section. From a higher resolution *Z*-contrast STEM image, Fig. 4(d), it is clearly observed that these intergrowths consist on a structure having an extra Cu–O chain layer inserted within the normal Y123 matrix, and hence they are identified as the well-known Y248 phase (see structure identification in inset of Fig. 4(d)).^26,27^

Occasionally, another type of intergrowth is also identified, indicated by arrows in Fig. 4(d), with two extra Cu–O chains being incorporated, resulting in a local composition of YBa_2_Cu_5_O_8_ (Y125) phase.^57^ These Y248 and Y125 intergrowths are surrounded by partial dislocations which generate localized strained regions^57^ reflected in an enhanced inhomogeneous strain (nanostrain) of the Y123 structure along the *c*-axis, as measured by the inhomogeneous integral breadth of (00*l*) Bragg peaks in the X-ray diffraction patterns (Fig. 3(d)).

In coherent epitaxial films, the lattice mismatch with the substrate leads to lattice deformation perpendicular to it,^47^ and this can be modified depending on the amount of misfit dislocations formed at the interface. In our ultrathin films misfit dislocations, previously observed in CSD Y123‖LAO thick films,^26,38^ do not form in the Y248 layers identified by TEM at the interface (Fig. 4(c)). Therefore, the Y123 layers embedded in the film can display a strong lattice expansion along *c*-axis, as it is experimentally demonstrated by X-ray diffraction (Fig. 3(a)).

It is not straightforward to understand why CSD Y123 films have the high concentration of Y248 intergrowths identified by STEM. Actually, the generation of these intergrowths within the Y123 matrix has been described previously as a mechanism to accommodate lattice deformations at interfaces.^10,58^ Therefore, our results suggest that the microstructural landscape of CSD Y123 ultrathin films is also driven by a release of the elastic energy associated to the lattice mismatch at the LAO interface.

#### c Concentration of intergrowths *versus* film thickness

A point of central interest is to investigate the evolution of the concentration of the intergrowths with film thickness. The analysis of the XRD patterns of the Y123 thin films did not show any Bragg peak associated to the Y248 phase, even if it was directly visible on the Z-contrast STEM images. As we have mentioned before, the Y248 intergrowths are characterized by a special structure having double Cu–O chains with a high concentration of defect clusters including two Cu vacancies decorated by three O vacancies which induce some additional disorder in the neighboring CuO_2_ planes and preserve the overall Y123 (1 : 2 : 3) cation stoichiometry.^27,30^

The disordered arrangement at the nanoscale of Y248 intergrowths perpendicularly to the *c*-axis will lead to a strong X-ray diffuse scattering which will decrease the coherent contribution to the corresponding (00*l*) X-ray diffraction intensity peaks of Y248, at the limit of becoming invisible.^59,60^ We can then have a rough estimation of the concentration of the Y248 intergrowths *via* the calculation of the integrated intensity of a Y123 (00*l*) Bragg peak and comparing them with that expected for a film of the same thickness having 100% of the volume with the Y123 phase. This estimation is based on two preconditions, one is that all the films are free of secondary phases after growth (Fig. 2(a)–(e)) and the other one is that we can use the CTA 250 nm Y123 films as a reference for a film having 100% of its volume as the Y123 structure (Fig. 4(b)).

**Fig. 5:**
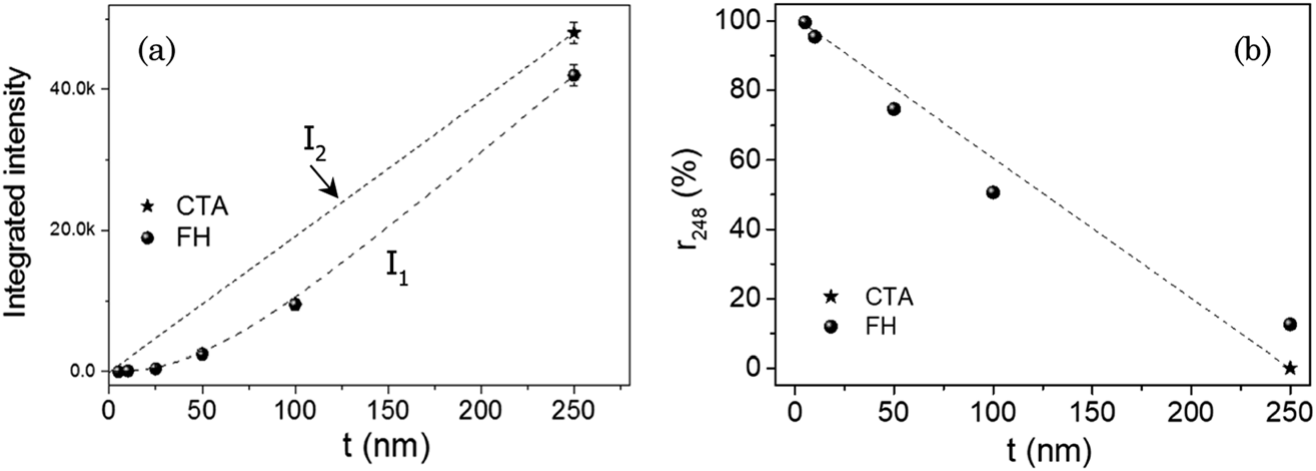
(a) Thickness dependence of the experimental integrated area *I*_1_ of (005) Bragg peaks in the XRD patterns of FH Y123 films shown in Fig. 3(a). This intensity is compared to the expected intensity in films with 100% volume of non-disordered Y123 phase *I*_2_. The reference for *I*_2_ corresponds to a Y123 CTA film with a thickness of 250 nm where no Y248 intergrowths are observed by STEM; (b) volume percentage of Y248 phase (*r*_Y248_) *versus* film thickness estimated from the observed differences *I*_2_–*I*_1_ in (a). Dashed lines are guides to eyes.

In Fig. 5(a) we present the integrated area *I*_1_ of the experimentally determined Y123 (005) Bragg peaks (see Fig. 2(b)), as a function of film thickness. We include in the same figure the intensity expected for non-disordered Y123 films *I*_2_, taking as a reference the intensity of a CTA 250 nm Y123 film where Y248 intergrowths are practically absent (linear decrease of intensity with thickness). It is clearly seen that the experimental intensities of the FH films (dotted line, *I*_1_) are well below the expected values for a 100% volume of Y123 films (solid line, *I*_2_). Then, taking into account that *I*_1_ is proportional to the volume of Y123 phase, we can estimate that the volume percentage of the Y248 phase *r*_Y248_ for the FH films at different film thicknesses corresponds roughly to:
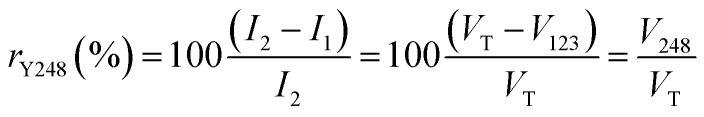
where for each thickness *V*_T_ is the total volume of the film, *V*_123_ is the volume occupied by the Y123 phase and *V*_248_ is the volume of the Y248 phase. The estimated evolution of *r*_Y248_ with film thickness is displayed in Fig. 5(b). Note that the values of *r*_Y248_ increase continuously with the decrease of film thickness. Especially, films with thickness ≤25 nm display very high *r*_Y248_ values (≥90%), evidencing the preponderance of the Y248 phase over that of Y123.

In conclusion, the pristine Y123 CSD ultrathin films grown by FH present a small percentage of the Y123 lattice structure, instead, a high density of the defective Y248 structure has been formed, making our films very rich in Cu and O vacancies in the double chains of the Y248 structure which extends now over the whole film thickness. This unique structural landscape enables us to investigate the superconducting behavior of the most common defect in CSD films, *i.e.* Y248 intergrowths.

### 3.2. Superconducting properties

Superconducting properties of the Y123 films have been investigated using isothermal and temperature dependent magnetization measurements. First of all, we should stress that, in agreement with the microstructural degradation mentioned before for Y123 ultrathin films grown on substrates with tensile misfit (*i.e.* STO), the low field ZFC (∼0.2 mT) magnetic susceptibility measurements showed smaller *T*_c_ values (see ESI†). Therefore, we will concentrate our attention on the superconducting properties of films grown on LAO substrates having a compressive misfit.

**Fig. 6:**
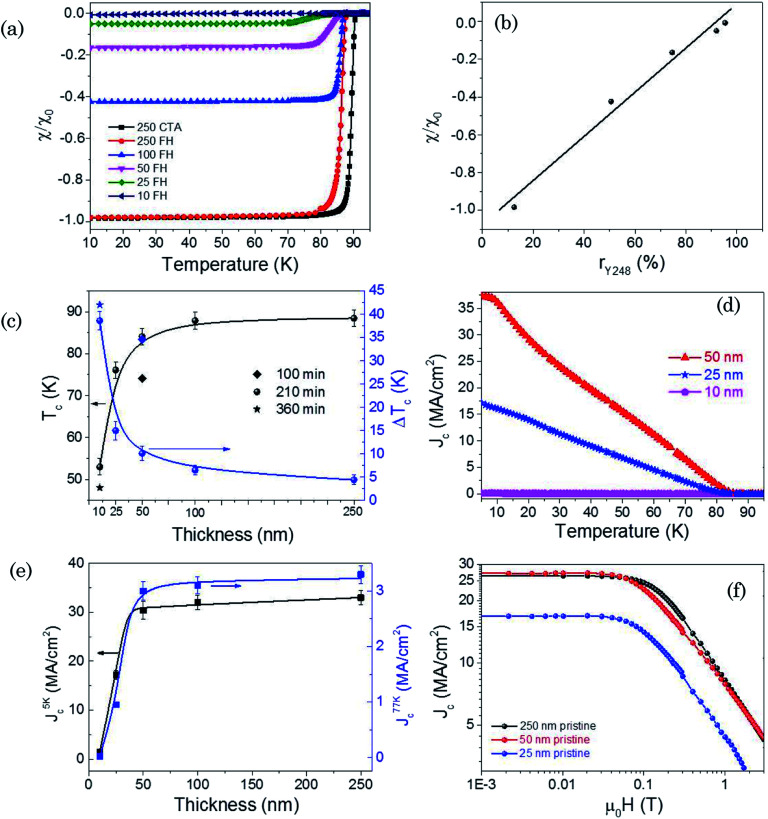
(a) Temperature dependence of magnetic susceptibility *χ*/*χ*_0_ measured at low magnetic field (0.2 mT) of Y123 films of different thicknesses and grown by flash heating or conventional thermal annealing (inset); (b) correlation of *χ*(0)/*χ*_0_ with the volume percentage of Y248 (*r*_Y248_) quoted in Fig. 4(b), *i.e.* the fraction of superconducting volume *versus* the estimated fraction of Y248 phase; (c) dependence of *T*_c_ and Δ*T*_c_ with film thickness. Inset: oxygenation times of the thin films; (d) temperature dependence of self-field critical current density *J*^sf^_c_(*T*) for films with different thickness (inset); (e) thickness dependence of *J*^sf^_c_(5 K) and *J*^sf^_c_(77 K) values. Dots account for the mean value of each while error bars account for the statistical distribution. (f) *J*_c_(*H*) dependence with magnetic field measured at 5 K for pristine Y123 films (star) with thickness of 25 nm (blue), 50 nm (red) and 250 (black). All the measurements were performed with *H*‖*c*.

In Fig. 6(a), we present the ZFC magnetic susceptibility *χ*(*T*) measurements of the Y123 films, measured at 0.2 mT, with thicknesses ranging from 10 nm to 250 nm. In the thin film approximation, the initial susceptibility of thin films with a disk shape can be written as *χ*_0_ = (8*R*/3π*t*), where *R* and *t* are the radius and thickness of the disk.^42,43^ Therefore, the normalized susceptibility *χ*(*T*)/*χ*_0_ is a measure of the shielding capacity of the thin films, *i.e.* for a full superconducting films we have *χ*(0)/*χ*_0_ = −1, while a decrease of this value is a measure of a reduced superconducting volume. Fig. 6(a) clearly indicates that while films with large thicknesses (∼250 nm) display a full superconducting shielding behavior, a progressive decrease of the superconducting volume occurs when the film thickness decreases. Fig. 6(b) shows that, actually, a close correlation exists between the decrease of the ratio *χ*(0)/*χ*_0_, *i.e.* the superconducting volume, and the volume percentage of the Y248 intergrowths, as estimated from X-ray diffraction, see *r*_Y248_ in Fig. 5(b). Our results show that extrapolation to full suppression of superconductivity occurs very close to the limit of 100% Y248, thus suggesting that this defective double layer structure has a non-superconducting behavior and it is intermixed with the remaining Y123 phase which is responsible of the observed superconducting behavior. The shielding currents have, therefore, a percolative behavior along the Y123 layered structure while the Y248 intergrowths would allow full flux penetration.

The evolution of the corresponding critical temperature (*T*_c_) and transition width (Δ*T*_c_) of the Y123 films with the total film thickness is shown in Fig. 6(c). It is noteworthy that *T*_c_ gradually decreases down to 50 nm film thickness, followed by a sudden drop at lower thicknesses. Similar behaviors have also been observed in vacuum deposited Y123 films^47,56^ or strained superlattices,^54,61^ even if the observed *T*_c_ decrease is more severe in the present case. We should note, as well, the obvious increase of Δ*T*_c_ which is very likely influenced by a decrease of the shielding efficiency of the percolating currents at smaller film thickness. This is consistent with the increase of the concentration of non-superconducting Y248 intergrowths, although an enhanced structural disorder, as revealed by the decrease of the out-of-plane texture quality and the increase of nanostrain (Fig. 2(b) and (d)), could also have some influence on Δ*T*_c_.

The dependence with the film thickness of self-field critical current density when *H*‖*c*, *J*^sf^_c_, calculated using the Bean model as indicated in section 2, is illustrated in Fig. 6(d) and (e). Using the thin film approximation of the Bean model to estimate *J*^sf^_c_(*T*) assumes, in the present case, that the macroscopic flux profile across the films is established in spite of the nanoscale inhomogeneous superconducting character of the films having a high concentration of Y248 volume where superconductivity is supressed. This mixed superconducting and non-superconducting microscopic structure was already previously tested in several sorts of superconductors, such as for instance superconducting foams,^62^ where the validity of establishing a critical state profile with an effective critical current density was assessed. A progressive degradation of *J*^sf^_c_(*T*) with the decrease of film thickness is also clearly identified here. In particular, the film with a thickness of 10 nm shows a practical absence of superconducting behavior at all temperatures. Fig. 6(e) displays the evolution, as a function of films thickness, of *J*^sf^_c_ values, both at 5 K (*J*^sf^_c_(5 K)) and 77 K (*J*^sf^_c_(77 K)). We observe that *J*^sf^_c_ keep constant values, *i.e. J*^sf^_c_(5 K) = 30.0 ± 2.0 MA cm^−2^ and *J*^sf^_c_(77 K) = 3.2 ± 0.2 MA cm^−2^, for films with thicknesses ranging from 50 nm to 250 nm. On the other hand, a strong tendency towards *J*^sf^_c_ degradation is clearly observed when the film thicknesses further decreases. We have confirmed the observed decrease of the critical currents in ultrathin films by investigating the isothermal magnetic field dependence *J*_c_(*H*) at 5 K (Fig. 6(f)). The sudden drop with thickness reduction of *J*^sf^_c_(*T*) and *J*_c_(*H*) values follows closely the observed decrease of *T*_c_ while the superconducting volume determined through magnetic susceptibility measurements has a steady decrease (Fig. 6(a) and (b)). Very likely the decrease of the inductively estimated *J*^sf^_c_ values of the remaining superconducting Y123 layers arise from a combined effect of a reduced superconducting order parameter (reduced *T*_c_) and from the geometrical effect of the non-superconducting volume in the films (Y248 intergrowths) which reduces the cross section of the percolating currents and so leads to reduced effective *J*^sf^_c_ values.

Finally, we should stress that several authors have previously reported that superconductivity can be either enhanced or degraded at interfaces in strained high temperature superconducting films due either to the in-plane strain with the substrate,^47,63^ to strain induced oxygen deficiency or to atomic disorder.^64,65^ For the purpose of assessing the role of oxygenation time on the superconducting performance of the ultrathin films, we have also analysed the influence of an extension of the oxygenation time from 100 min to 360 min in the superconducting properties of these films. Note that in our case further increase of the oxygenation time up to 360 min presents very little changes of *T*_c_ or Δ*T*_c_, see Fig. 6(c). This suggests that superconductivity quenching in the ultrathin CSD Y123 films is very likely not the result of oxygen deficiency, in agreement with our analysis of the increase of *c*-axis parameter (Fig. 3(a)).^49,66^ It's, however, well known that oxygen kinetics in oxides may be strongly influenced by local strain and surface barriers and so we cannot fully disregard that some oxygen deficiency remains.^67–69^

In summary, we have provided evidence for the suppression of superconductivity at the nanoscale in the Y248 intergrowths, although the microscopic origin of this behavior remains an open issue. As it was recently reported, the Cu–O double chains of Y248 intergrowths include a high concentration of defective clusters consisting of two Cu vacancies decorated by three O vacancies.^27,30^ These defects also were shown to lead to the formation of a nanoscale ferromagnetic (superparamagnetic) behavior, a highly distorted Y123 matrix around them, including apical oxygen vacancies, and a modified electronic structure in the neighboring CuO_2_ planes, as detected by EELS and XMCD analysis.^27,29,30^ Very likely quenching of the Cooper pair formation occurs at the nanoscale in the full volume of the Y248 intergrowths.^10,70^ We suggest now that the double chains of the defective Y248 structure, and the CuO_2_ planes next to them, have an absence or a very perturbed superconducting behavior. The observed progressive reduction of the superconducting volume with film thickness decrease is then associated to an enhanced volume percentage of the Y248 intergrowths. The decrease of *T*_c_, on the other hand, should reflect the lattice deformation of the Y123 layers remaining in the films.

## 4. Conclusions

We report an investigation of the influence of the micro/nanostructure changes of chemical solution deposited Y123 films varying thicknesses, down to 5 nm, and their consequences on the superconducting properties. Ultrathin Y123 epitaxial films have been successfully grown on LaAlO_3_ substrates based on optimized crystallization conditions. Detailed microstructural investigations of the Y123 ultrathin films by means of XRD and STEM have demonstrated that these thin films are epitaxial with an increased concentration of Y248 intergrowths modifying the films nanostructure when the film thickness decreases, even if the overall Y:2Ba:3Cu stoichiometry is preserved. The progressive increase of the volume percentage of Y248 intergrowths when the film thickness decreases has been closely correlated with a corresponding shrinking of the superconducting volume as measured by low field magnetic shielding, thus suggesting a suppression of the superconducting behavior at the nanoscale.

Defective Y248 intergrowths include a high concentration of Cu and O vacancies and the present work has shown that this defective structure is strong enough to suppress its superconducting behavior thus making to behave fundamentally different from the stoichiometric Y248 phase displaying *T*_c_ values in the range of ∼80 K. This conclusion gives support to the idea that defective Y248 intergrowths play a key role as artificial pinning centers of vortices in Y123 nanocomposite films and coated conductors.

## Conflicts of interest

The authors declare no competing interest

## Supplementary Material

NA-002-D0NA00456A-s001
