# Supplementary material for: Suppression of superconductivity at the nanoscale in chemical solution derived YBa2Cu3O7−δ thin films with defective Y2Ba4Cu8O16 intergrowths
Source: Nanoscale Adv. 2020 Jun 24;2(8):3384–93. doi: 10.1039/d0na00456a (PMC9418937; doi:10.1039/d0na00456a)
Supplement: NA-002-D0NA00456A-s001 [file NA-002-D0NA00456A-s001.pdf]

Electronic Supplementary Material (ESI) for Nanoscale Advances.
This journal is © The Royal Society of Chemistry 2020

SUPPORTING INFORMATION

Suppression of superconductivity at the nanoscale in chemical solution derived $YBa_2Cu_3O_{7-\delta}$ thin films with defective $Y_2Ba_4Cu_8O_{16}$ intergrowths

Ziliang Li, Mariona Coll, Bernat Mundet, Anna Palau, Teresa Puig, Xavier Obradors*

Institut de Ciència de Materials de Barcelona, CSIC, Campus de la UAB, 08193 Bellaterra, Catalonia, Spain

S1. Thermal profile

In Figure S1 we present a sketch showing the general profiles of the thermal treatment for crystallization. The crystallization thermal processes are defined in the form of, for instance, Flash Heating (FH) T ºC (t_1 + t_2), denoting the films were flash heated to the growth temperature (T ºC) and then annealed in humid and dry gas atmosphere for t_1 and t_2 min, respectively. Conventional Thermal Annealing (CTA) corresponds to heating at a rate of 25ºC/min. FH process has been described previously in detail [1], it corresponds to a heating rate in the range of ~750 ºC/min, i.e. ~30 times faster than CTA.

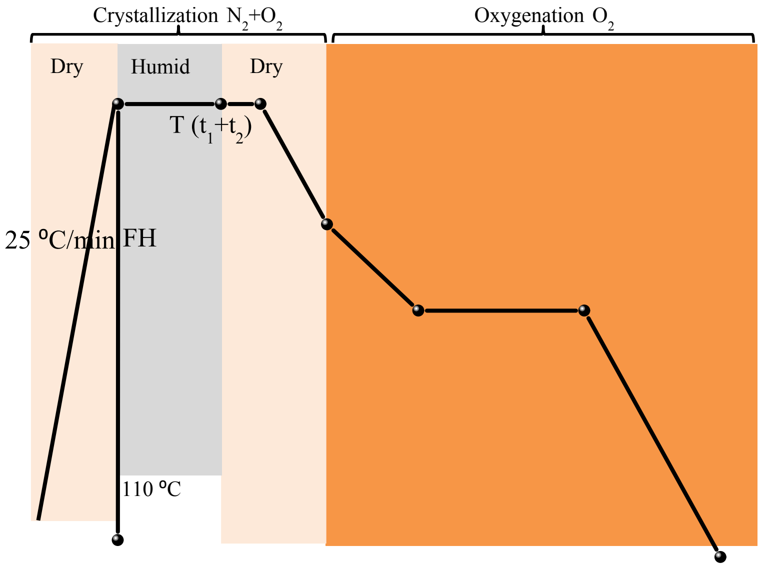

Figure S1: Schematic thermal profile applied for the formation of Y123 ultrathin films: Flash Heating and Conventional Thermal Annealing.

S2. The influence of heating rate: Conventional thermal annealing vs Flash Heating

The TFA solutions with a total metal concentration of 0.06 M were deposited on LAO substrate and grown from the Conventional Thermal Annealing (CTA) 810 ºC (20+10) and FH 810 ºC (20+10) process, respectively.

Figure S2 shows Z-contrast images of 10 nm films grown from (a)-(b) CTA 810 ºC (20+10) and (c)-(d) FH 810 ºC (20+10). The film grown from the CTA process is discontinuous, identifying the dewetting effect [2], forming Y123 islands (Figure S2 (a)). Higher resolution STEM image indicates that the film displays inhomogeneous distribution of film thickness (Figure S2 (b)). The film grown from the FH process appears as a continuous Y123 layer (Figure S2 (c)) with uniformly distributed film thickness being the mean value of ~ 10 nm (Figure S2 (d)). This observation indicates that the FH process is an effective pathway for achieving continuous ultrathin films.

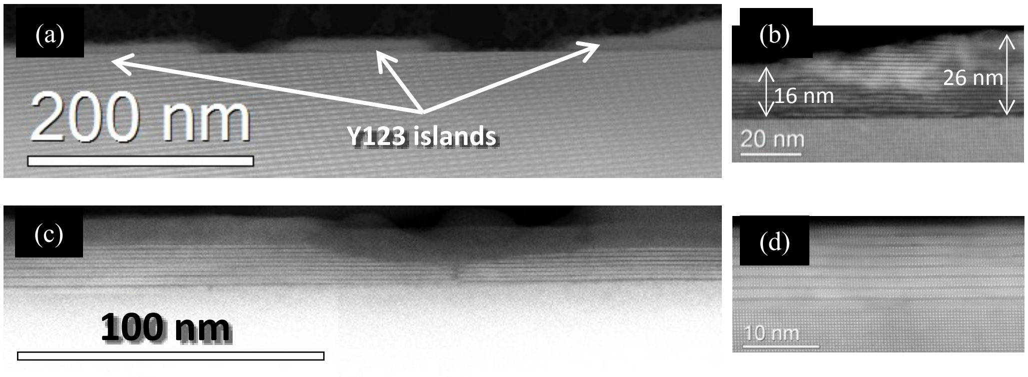

Figure S2: Cross section Z-contrast STEM images of 10 nm Y123 ultrathin films grown from (a)-(b) CTA 810 ºC (20+10) and (c)-(d) FH 810 ºC (20+10).

S3. The influence of thermal annealing time

In order to disclose the possibility of obtaining continuous Y123 ultrathin films by tuning growth kinetics, the growth of 50 nm Y123 films was carried out using FH 810 ºC process annealed for (20+10) min, (20+30) min and (60+30) min. The films were investigated by means of SEM observation, as shown in Figure S3. As it can be clearly seen in the micrographs, the film grown from the FH 810 ºC (20+10) process has a dense, homogeneous surface with negligible amount of precipitates, as shown in Figure S3 (a). Further increase of annealing time to (20+30) and (60+30) min induces severe film dewetting (Figure S3 (b) and (c)), indicating that the fully converted ultrathin films obtained from the FH 810 ºC (20+10) process in a metastable state where a new equilibrium state can be reached via a slow atom diffusion, similar phenomenon occurs for the films with larger thickness (≥ 150 nm) in the previous work based on the MOD approach [2].

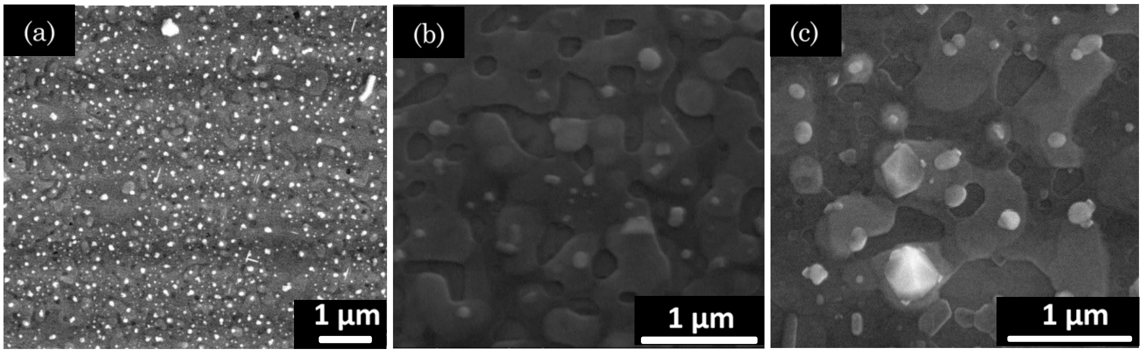

Figure S3: SEM micrographs of 50 nm Y123 ultrathin films grown following the same conditions (FH 810 ºC) except for the annealing time, i.e. (a) (20+10) min, (b) (20+30) min and (c) (60+30) min.

S4. The influence of crystallization temperature

We have also investigated the influence of growth temperatures on the microstructure and superconducting properties of Y123 ultrathin films, where three growth processes were applied, i.e. FH 750 °C (20+10), FH 810 °C (20+10) and FH 830 °C (20+10). Surface morphological investigation via SEM observation indicates that the film grown by the FH 750 °C (20+10) process (Figure S4 (a)) displays high density of large pores and small amount of randomly oriented grains. While the film grown from FH 810 °C (20+10) process presents a homogeneous surface with a small amount of precipitates and a negligible concentration of pores, as shown in Figures S4 (b). In contrast, the surface of the film grown from the FH 830 °C (20+10) process appear to be severely inhomogeneous with some anomalous bright regions appearing in the SEM image, as shown in Figure S4 (c). These regions were identified by EDX to correspond to LAO and so they have been identified as dewetting.

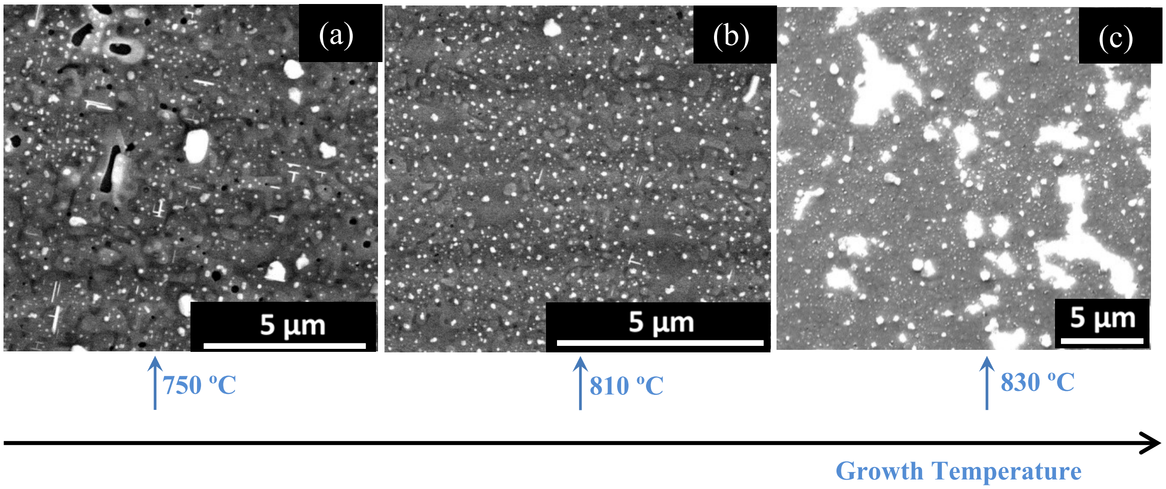

Figure S4: SEM micrographs of 50 nm TFA-Y123 films grown following the growth process of (a) FH 750 °C (20+10); (b) FH 810 °C (20+10) and (c) FH 830 °C (20+10).

A higher amplification SEM micrograph focusing on one bright area (from Figure S4 (c)) is presented in Figure S5 (a). As can be clearly seen, this bright region presents a pronounced dewetting character, where an anomalous pit appears within the layer with apparent lateral faces being in a wandering manner. It is worth noting that some cylindrical-like grains distribute within this region. EDX analysis demonstrates the presence of La, Al and O, which are in good accordance with the components of the single crystal substrate: $LaAlO_3$. In contrast, the peaks of Y123 components (i.e. Y, Ba and Cu) are negligible, indicating a full exposure of LAO. Therefore, it is reasonable to conclude that a full migration of the Y123 film occurs in these regions. It is observed in the high resolution X-ray diffraction (HRXRD) patterns (Figure S5 (b)) that besides the LAO (l00) Bragg reflections, it is also appear the (00l) oriented Y123 reflections, indicating c-axis preferred orientation of the Y123. However, intermediate phases such as $Y_2Cu_2O_5$ (Y225) have also been detected for the film grown at 830 ºC.

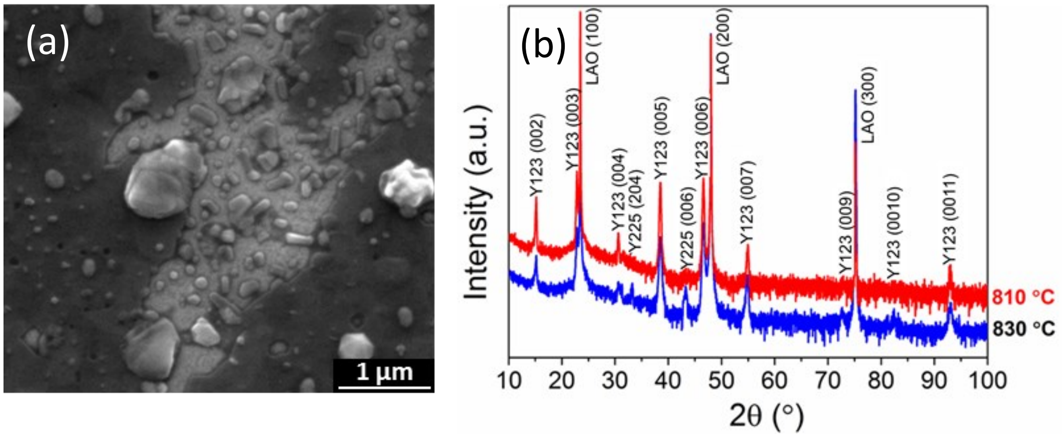

Figure S5 (a) a higher resolution SEM micrograph focusing on the squared region in Figure S4 (c); (b) HRXRD θ-2θ scans of 50 nm Y123 films grown from FH 810 °C (20+10) (red) and FH 830 °C (20+10) (blue), respectively.

S5. The influence of misfit strain from substrate

Figures S6 (a) and (b) display SEM micrographs of 50 nm Y123 films grown from the FH 810 ºC (20+10) process on LAO and STO single crystals, respectively, where the influence of mismatch strain on the film surface morphology can be clearly observed. The film grown on LAO displays a homogeneous surface with negligible porosity; see Figure S6 (a). The 50 nm Y123//STO film presents a high amount of holes. This result demonstrates that film grown on STO single crystal presents a higher tendency to dewet than with LAO substrate, in agreement with the previous prediction [2] that the tensile strain generated associated with the Y123/STO lattice misfit shows higher tendency for the formation of dewetting regions compared with the compressive strain. It is also remarkable that we observed a small fraction of randomly

oriented grains distributed on the film surface, consistently with the result previously detected from the XRD technique.

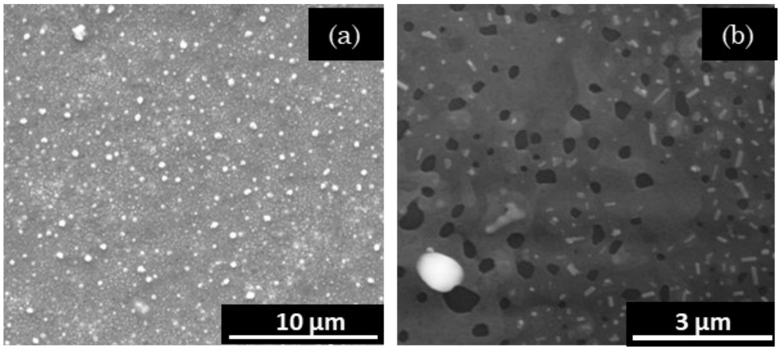

Figure S6 SEM planar morphology of (a) 50 nm Y123//LAO and (b) 50 nm Y123//STO film grown from the optimized FH 810 ℃ (20+10) process.

S6. Surface morphology of ultrathin Y123 films

SEM images of films grown on LAO with thickness ranging from 50-5 nm (see Figures S7 (a)-(c)) present a fairly dense and homogeneous surface morphology while that with thickness of 2.5 nm (see Figure S7 (d)) displays a very pronounced inhomogeneous characteristic with many recessed areas in an irregular manner. Therefore, we conclude that continuous Y123 single crystal film with thickness down to 5 nm is achieved based on the CSD FH route. This achievement enables to further investigate the microstructural and superconducting performance of ultrathin films. The bright particles observed in all the SEM images correspond to secondary phases segregated at the surface.

Additional quantitative investigation of surface morphological evolution with film thickness has also been conducted by AFM analysis (see typical images in Figure S8). The results disclose that secondary phases segregated at the surface are formed (see also SEM images in Figure S7). These surface impurities are sometimes observed in CSD Y123 films and they correspond to $Y_2Cu_2O_5$ or $BaCuO_2$ phases. They are usually associated to slight non-stoichiometry of the initial solutions or uncompleted reaction to form Y123. The increase of film roughness in thicker films is associated to a higher concentration of the secondary phases at the surface of the films. We should note that the CSD films after the pyrolysis have a low surface roughness (R_{rms} = ~1.55 nm) and so the increase of roughness originates during the final transformation to Y123.

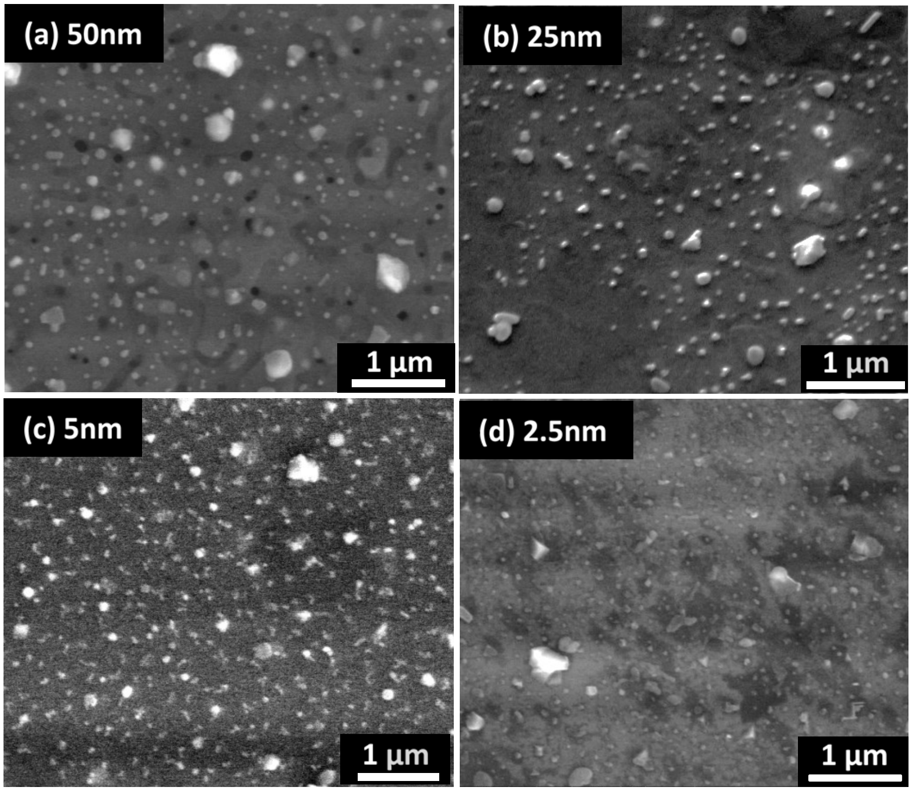

Figure S7 SEM images of pristine Y123 ultrathin films with thicknesses of (a) 50 nm, (b) 25 nm, (c) 5 nm and (d) 2.5 nm.

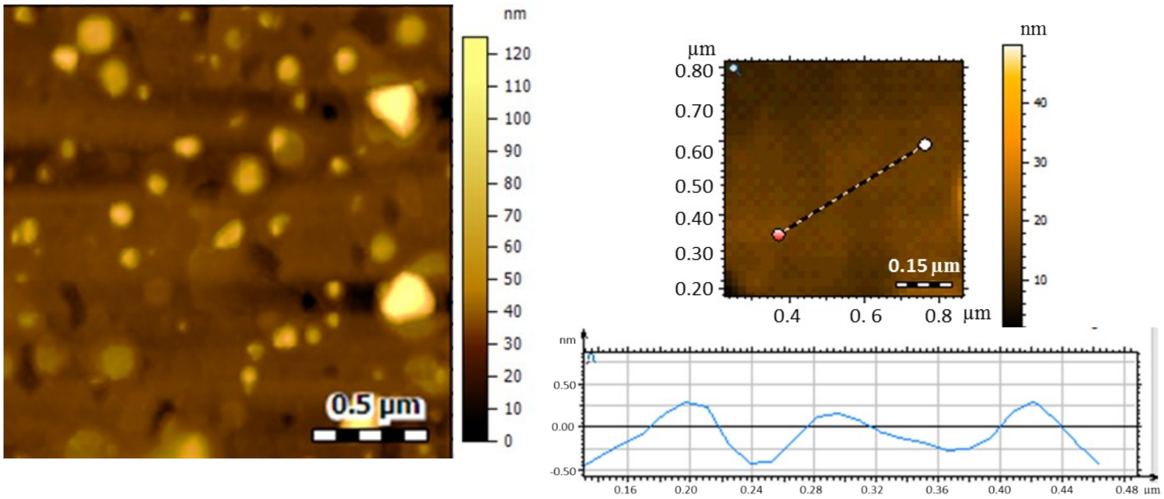

Figure S8 AFM images of (a) a 10 nm and (b) a 5 nm Y123//LAO film grown from the optimized FH 810 ºC (20+10) process. Image (a) display typical secondary phases segregated at the surface while image (b) display a line scan taken in a region of the surface where the impurities at the surface are avoided and so it reflects the homogeneity of the thickness of the Y123 film.

S7. Superconducting properties

The plots of Low Field Cooled (LFC, 2 Oe) magnetization dependence with temperature of the films are presented in Figure S9. It is clear seen that the 50 nm Y123 film grown on STO shows degraded T_c compared with the one on LAO, and it is consistent with the previous observation [3] which has attributed the T_c difference to the substrate-induced strain effects. Moreover, for the film grown on STO, we observe a broadened transition width (ΔT_c) compared with the one on LAO. This broadening can be attributed to dewetting regions induced low current percolation.

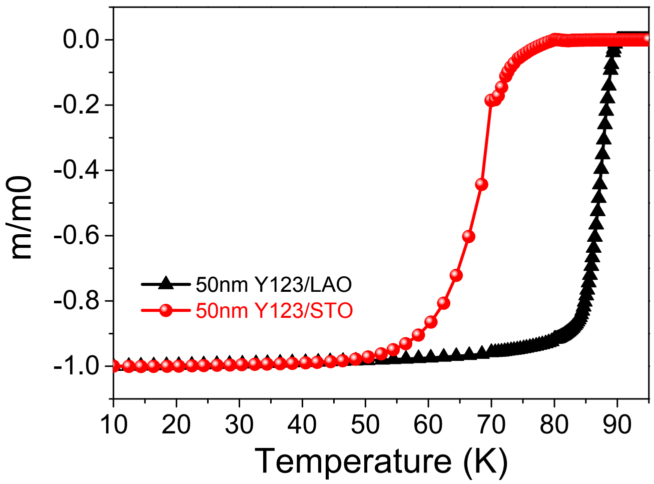

Figure S9 Temperature dependence of Zero Field Cooled (ZFC) magnetization of 50 nm Y123 films grown on top of LAO (black triangle) and STO (red ball) substrates from the FH 810 °C (20+10) process.

The critical current densities of the different thin films were estimated from the recorded magnetization hysteresis loops, such as that shown in Figure S10, using the Bean model approximation to thin films, $J_c(H,T)=3M(H,T)/R$, where R is the effective radius of the sample. The hysteretic magnetization is $M = (m_p-m_n)/V$, calculated from the positive and negative values of magnetic moment and V is the volume of the film, (Figure S10). M(H) was measured only in the positive side of the external magnetic field.

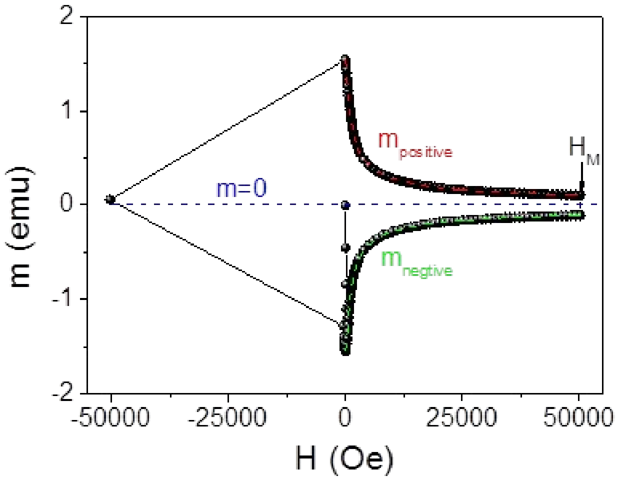

Figure S10 Initial data obtained from the SQUID measurement for a typical Y123 film of magnetic moment as a function of magnetic field measured at 5K, from where the critical current densities can be estimated following the Equation 1-1

References

(1) Li, Z.; Coll, M.; Mundet, B.; Palau, A.; Puig, T.; Obradors, X. Accelerated growth by flash heating of high critical current Trifluoroacetate solution derived epitaxial superconducting $YBa_2Cu_3O_7$ films. *Journal of Materials Chemistry C* **2019,** *7*, 4748.
(2) Coll, M.; Gazquez, J.; Pomar, A.; Puig, T.; Sandiumenge, F.; Obradors, X. Stress-induced spontaneous dewetting of heteroepitaxial $YBa_2Cu_3O_7$ thin films. *Physical Review B* **2006,** *73* (7), 075420.
(3) Zhai, H. Y.; Chu, W. K. Effect of interfacial strain on critical temperature of $YBa_2Cu_3O_{7-\delta}$ thin films. *Applied Physics Letters* **2000,** *76* (23), 3469-3471.
